# Transient third nerve palsy after percutaneous coronary intervention – A case report

**DOI:** 10.22336/rjo.2026.22

**Published:** 2026

**Authors:** Divya Trivedi, Vinita Ramnani, Madhuri Nagori, Rahul Jain, Sakshi Ramnani

**Affiliations:** 1 Department of Ophthalmology, Sadguru Sankalp Netra Chikitsalaya, Anandpur, Vidisha, India; 2 Department of Ophthalmology, Bansal Hospital, Bhopal, India; 3 Department of Cardiology, Bansal Hospital, Bhopal, India; 4 Department of Radiology, Bansal Hospital, Bhopal, India; 5 Shri Bhagwan Mahavir Vitreo Retinal Services, Sankara Nethralaya, Chennai, India

**Keywords:** neurological complications, oculomotor nerve palsy, diplopia, coronary angioplasty, PTCA = Percutaneous Transluminal Coronary Angioplasty, MRI = Magnetic Resonance Imaging, MRA = Magnetic Resonance Angiography, ECG = Electrocardiogram, LAD = Left Anterior Descending, RCA = Right Coronary Artery, CVA = Cerebro-Vascular Accident, CAD = Coronary Artery Disease, ADC = Apparent Diffusion Coefficient

## Abstract

Oculomotor nerve palsy is a rare complication of interventional cardiac procedures. We report a case of a 64-year-old male who developed diplopia after he underwent a percutaneous transluminal coronary angioplasty (PTCA) for double-vessel disease. Ocular examination revealed partial third nerve palsy in his right eye. His anterior and posterior segment examinations were within normal limits. Brain MRI revealed a hyperacute infarct in the right paramedian region of the superior colliculus. MR angiography displayed stenosis in his right internal carotid artery in the cervical region. He was managed conservatively with anticoagulants and statins, and his ocular movements gradually improved without any active ocular intervention. This case highlights the potential risk of neurological complications following an invasive cardiac procedure.

## Introduction

Neurological complications after cardiac procedures are a rare occurrence [1]. This may clinically manifest as blurring of vision, diplopia, nystagmus, cortical blindness, or internuclear ophthalmoplegia [2]. The exact pathophysiology is unclear, but it has been proposed to result from cerebral embolism originating predominantly from large atherosclerotic vessels [2]. We report a case of an elderly male who developed a partial third nerve palsy within days of undergoing a percutaneous transluminal coronary angioplasty (PTCA).

## Case report

**Fig. 1:**
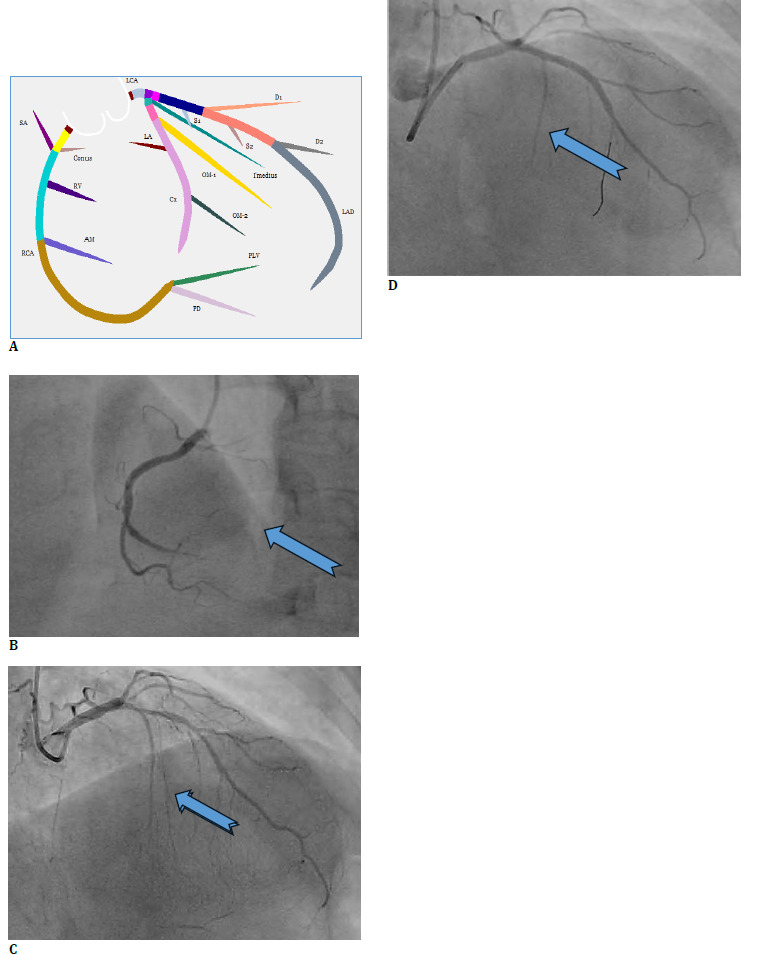
(**A**) Coronary angiography depicting cardiac vasculature; (**B**) Distal right coronary artery (RCA) 100% block (blue arrow); (**C**) Left anterior descending artery (LAD) 90% block (blue arrow); (D) Post-PTCA stent in LAD (blue arrow)

A 64-year-old male presented with a sudden onset of chest pain and was admitted for evaluation in the cardiology department. He was a known diabetic and hypertensive, had a history of a cerebrovascular accident, and had coronary artery disease with an inferior wall myocardial infarction. His electrocardiogram (ECG) showed ST-T changes, following which he was posted for a coronary angiography. Angiogram revealed double-vessel disease, with severe obstructions in his left anterior descending artery (LAD) (90% in proximal LAD; 50% in mid-LAD) and right coronary artery (RCA) (100%) (**Fig. 1A-D**). In view of the above findings, he underwent a percutaneous transluminal coronary angioplasty (PTCA). The procedure was uneventful, and he was recovering well.

**Fig. 2:**
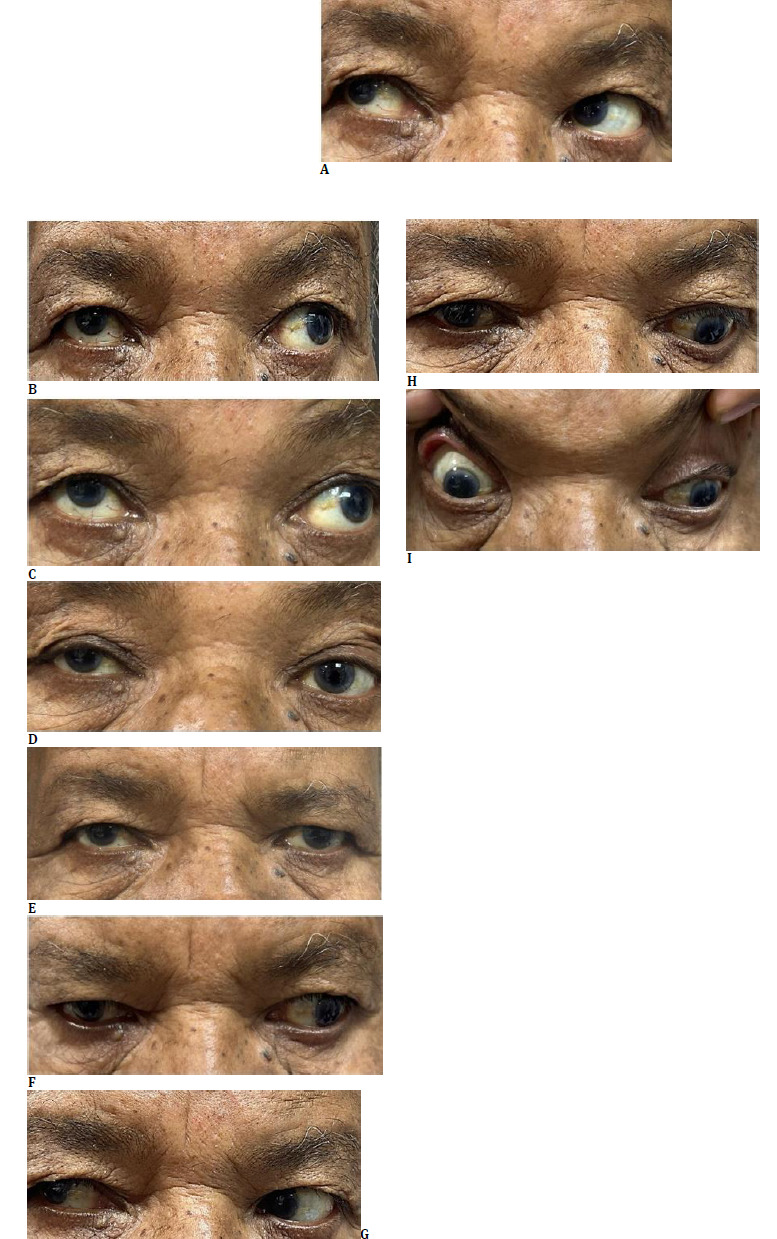
**A-I** Extraocular movements in the nine cardinal gazes, suggestive of right oculomotor nerve palsy

**Fig. 3:**
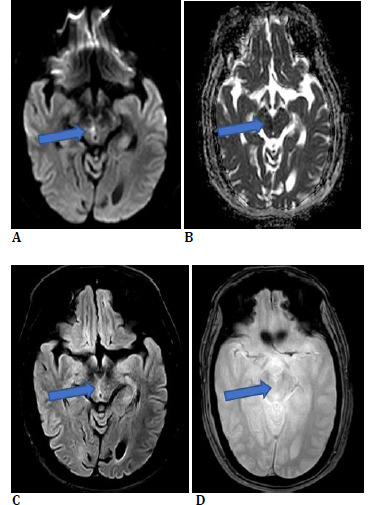
MRI brain at the level of upper mid brain (superior colliculus) showing (**A**) tiny focus of mild diffusion restriction on diffusion weighted imaging (**B**) with corresponding low ADC value (**C**), which shows FLAIR mismatch suggestive of hyperacute infarct at right paramedian location; (**D**) No blooming on gradient echo images

However, during his postoperative stay in the hospital, he experienced two episodes of vertigo and blurred vision. He had no accompanying dysphagia, dysphasia, or motor and/or sensory abnormalities in his extremities. On bedside examination, his vision was recorded as counting fingers at 3 meters in both his eyes, with normal pupillary reflexes, and no ptosis. Fundus examination was unremarkable. His extraocular movements demonstrated a limitation in adduction, supraduction, and infra-duction with normal abduction in his right eye, while his left eye displayed exaggerated abduction (**Fig. 2A-I**). Both pupils reacted briskly to light, and cranial nerve examination was normal. This was suggestive of a partial third nerve palsy in his right eye. Magnetic resonance imaging (MRI) of the brain was suggestive of a hyperacute infarct in the right paramedian region of the superior colliculus (**Fig. 3A-D**). Magnetic resonance angiography (MRA) revealed 20-30% luminal narrowing in the region of his right cervical internal carotid artery.

He was managed with dual antiplatelet drugs, statins, and beta-blockers. There was a gradual improvement in his ocular symptoms without the need for any intervention. On examination in the eye department, his best corrected visual acuity was 6/9, N9 in the right eye, and 6/6, N6 in the left eye. Slit lamp bio-microscopy showed an immature cataract in his right eye and pseudophakia in his left eye. Fundus examination was within normal limits, and his pupillary reflexes were intact. His extraocular movements regained a normal range of motion with no limitation. He was discharged after three days and was aggressively counseled about the risk factors precipitating his condition.

## Discussion

Coronary angiography and coronary angioplasty are universally accepted as standard procedures in the management of coronary artery disease. However, due to the invasive nature of these procedures, they carry a small risk of complications, including arrhythmias, reactions to contrast agents, and vascular and hemodynamic problems [3]. Neurological complications arising from interventional cardiac procedures are extremely rare, with an incidence of 0.2-0.4% [4]. Embolisms have been implicated in the pathogenesis of these issues. They may originate from atheromatous plaques, thrombus formation at the catheter tip, air emboli, and, very rarely, from foreign materials from the catheter and/or guide wire [4]. Such thromboembolic episodes may result in end-organ infarction, especially in the brain, kidneys, and eyes [5].

Elderly females with diabetes, hypertension, and deranged cholesterol levels appear to be at a higher risk of developing such complications. Larger catheters and a longer procedure duration are also associated with higher risks of adverse effects [6], regardless of the route of cardiac catheterization [7]. The vertebrobasilar circulation is preferentially affected, resulting in ocular symptoms such as blurring of vision, diplopia, cortical blindness, and internuclear ophthalmoplegia [7]. Our patient had all the known risk factors – elderly, diabetic, hypertensive, with a prior history of CVA and CAD – all predisposing him to transient vasospasm and emboli, and he responded well to aspirin and statin therapy.

Neuroimaging is crucial to clinch the diagnosis in these cases [8]. Given the embolic nature of most of these cases, they must be started on anticoagulants and/or statins, as per the treating cardiologists [9]. Similar cases, albeit infrequently, have been reported in the literature. Liu and coworkers documented two cases with bilateral third nerve palsy after cardiac procedures [10]. Biller and colleagues described a case of bilateral internuclear ophthalmoplegia, retraction nystagmus, and somnolence resulting from a dorsal tegmental infarction following angioplasty [11]. Along the same lines, Mohammadian M and Damati A reported a case of mild left-sided ptosis, binocular diplopia, and partial impairment of adduction in the left eye following coronary angiography. MRI brain revealed a midbrain tegmental infarction. Mihaescu et al. reported a case of bilateral ptosis, upgaze paresis, and internuclear ophthalmoplegia resulting from a periaqueductal grey matter infarction following cardiac catheterization [12].

## Conclusion

Oculomotor nerve palsy occurring after an invasive cardiac procedure is a rare but alarming complication. Though it often runs a benign course, it must be viewed with caution. Prompt neurological imaging and management in conjunction with the cardiology team can improve outcomes.

## Presentation at any previous meetings

Nil.
